# Year 2024: Trends towards Multi-[tasks, languages, models] and Large Adoption of LLMs

**DOI:** 10.1055/s-0046-1816633

**Published:** 2026-11-03

**Authors:** Cyril Grouin, Natalia Grabar

**Affiliations:** 1 Université Paris-Saclay, CNRS, LISN, 91400 OrsayFrance; 2 CNRS, Univ. Lille, UMR 8163 - STL - Savoirs Textes Langage, F-59000 LilleFrance

**Keywords:** Biomedical Natural Language Processing, Topics, Issues, Best Papers, 2024

## Abstract

**Objectives** : This synopsis gives insights into scientific publications from 2024 in Natural Language Processing (NLP) for the biomedical domain. We present the process we followed to identify candidates for NLP's best papers and the three best papers of this year. We also analyze the current trends in the 2024 NLP-related publications.

**Methods** : We queried the Medline database and refined the outputs through automatic scoring. We then manually preselected publications to review and selected candidate papers through an adjudication process. External reviewers assessed the interest of those 14 candidates. At last, the section editors chose the best NLP papers.

**Results** : We collected 2,781 papers published in 2024, of which three were the best and selected as part of the NLP chapter. The best papers address manual annotation strategies in clinical data, claim verification in scientific literature, and generation of explanations for diagnoses in clinical cases.

**Conclusion** : Trends from 2024 correspond to: i) multi* frameworks, in which several languages, tasks or models are addressed within the same experiments, which is due to the availability of language models and datasets; ii) intensive exploitation of clinical data and emergence of newspapers as yet another source of information to be processed; iii)increasing number of publications issued from China and covering Chinese data, even if the processing of English data remains the most frequent; and iv) large adoption of large language models (LLMs) for various tasks.

## 1. Introduction

Natural Language Processing (NLP) aims to provide methods, tools, and resources designed to mine textual and narrative documents and enable access to the information they convey [ 1 ]. While human languages are complex (for example, for a human, learning a human language requires many years to be fluent), the importance of using NLP approaches to mine documents produced by humans has been pointed out for a long time [ 2 ]. In this synopsis, we present the selection process applied this year and then analyze some publications' content.

## 2. Best Papers Selection Process

### 2.1 Automatic Extraction and Scoring

#### 2.1.1 Description

To identify the papers published during 2024 in the field of NLP for the biomedical domain, we queried the Medline 1 database, specifically dedicated to the biomedical domain. Contrary to previous years when we also queried the ACL Anthology allowing for publications in major NLP conferences, due to the high number of publications to process for 2024 (2,781 entries), we decided to only focus on the Medline database. In addition, we also noticed that no candidates from the ACL Anthology was kept in the previous editions.

The query we used (left of Figure 1 ) is voluntarily basic to bring back the maximum number of candidates. It consists of searching all papers published in English in 2024, having an abstract, and indexed in terms of “clinical language processing”, or “medical language processing”, or “natural language processing”. As of January 17, 2025, we collected 2,781 entries, much more than all previous years (2,114 entries for 2023; 1,670 for 2022; 1,204 for 2021; 739 for 2020; 767 for 2019; and only 435 for 2018). To refine the selection of the candidate's best papers and to identify scientific papers valuable for the NLP community, we defined an automatic scoring for each candidate, based on four main criteria:

**Figure 1.**
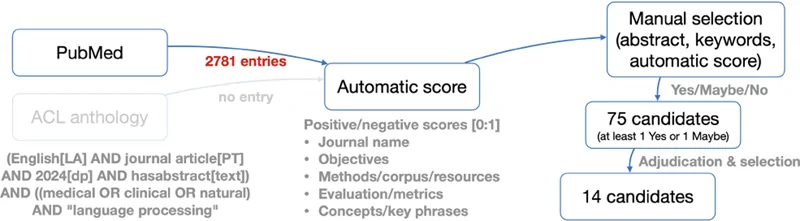
Process used to extract biomedical NLP publications from 2024 and the selection of best paper candidates

- Journal name. We defined a list of journals where the NLP community usually publishes its articles (e.g., BMC Bioinformatics, BMC Med Inform Decis Mak, Int J Med Inform, J Am Med Inform Assoc, J Biomed Inform, J Biomed Semantics, etc.) and journals where we do not publish and may be confusing or bad venues (e.g., Brain, Phoniatrics, Multimed, Neural Netw, Sensors, Speech Lang, etc.);
- Methods, corpora, resources used in the paper. We increase the score if codes, data, and models are proposed in an open-access mode;
- Type of evaluation and metrics used. As an example, the AUC (area under the curve) metric is generally not used in the NLP community (for which recall, precision, and F1-score are mostly used), but is used by machine-learning researchers or new artificial intelligence (AI) users;

Particular concepts or key-phrases used in the abstracts. We defined a list of concepts and key-phrases the NLP community is using (natural language processing, NLP, named entity recognition, NER, part-of-speech, POS, electronic health record, EHR, UMLS, annotated data, etc.) and concepts or key-phrases which are confusing and not related to NLP, even if related to language (word processing, language production, language comprehension, verbal intelligence, cerebral, etc.). Specifically, we consider that phrases like “using natural language processing”, “using NLP” and “perform a Natural Language Processing analysis” are not associated with innovative NLP work.

We also excluded yearbooks and survey papers since they do not fit the criteria for best paper candidates. For each of the 2,781 candidate papers, the final score ranged from 0 to 1 (cf. Figure 2 , dark green for 2024 in comparison to the year 2023 in light green).

**Figure 2.**
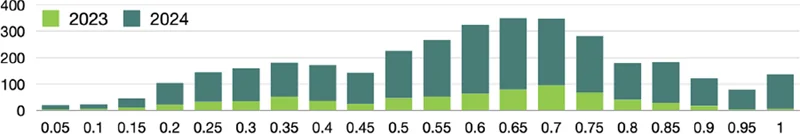
Distribution of papers according to the filter scores for years 2023 (light green) and 2024 (dark green).

#### 2.1.2 Analysis

Figure 3 shows the proportion (percentage) of articles per year returned by our Medline query, according to the country of affiliation of the first author, for four countries (USA, Canada, China, Australia), and two country groupings (Europe 2 and other countries). None of the countries in the “other countries” category is really emerging (India, Japan, Russia), apart from a few peaks in certain years. Their representation is similar to that of Australia and Canada, but with lower values. The diagram on the left shows a logarithmic representation of the curves for each country or group of countries. The diagram on the right normalizes this representation to 100% per year.

**Figure 3.**
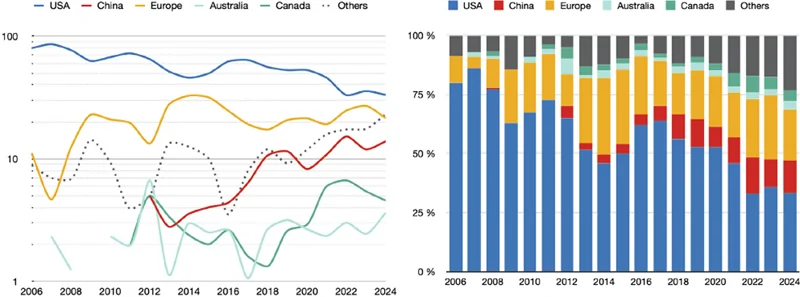
Percentage of articles published per year by country of affiliation of the first author (USA, China, Europe, Australia, Canada, other countries). Representation in curves on a logarithmic scale (left), normalized representation in bars (right).

The main findings are the following:

- Although this is not shown in the figure due to the percentage representation, authors from each country publish more each year;
- The number of publications from countries other than those listed is rising steadily, to account for almost a quarter of all articles published in 2024 (gray bar in the right part of Figure 3 );
- Until 2020, the USA were concerned by more than half of the publications with a first author affiliated within an institution from the USA. Yet, starting from 2021, the USA become represented by the first author in around a third of the published papers. The blue line in the figure is slowly decreasing, representing this lower representation. Conversely, the representation of China in the first authors of published papers is growing each year, and the red line in the figure is thus increasing and is closer to that of the USA while European countries as a whole (yellow) are tending to maintain their levels;
- The number of publications from countries other than those listed is rising steadily, to account for almost a quarter of all articles published in 2024 (gray bar in the right part of Figure 3 ).

### 2.2 Human Selection

Based on the automatic scores we computed (Section 2.1.1), each section editor quickly read the abstracts of articles with scores above 0.5 to determine whether the article in question was a good candidate for the best papers (label “yes”), perhaps a candidate (label “maybe”) or not a good candidate (label “no”). Based on the choices labeled “yes” or “maybe” by at least one of the two section editors, we obtained a list of 75 candidates. This list was then narrowed down to fourteen candidates through a bidding process between the two section editors, based in particular on the topics covered by the articles and the interest these articles might have for biomedical NLP. These articles are divided into the following main themes:

- synthetic data production [ 3 ], data annotation [ 4 , 5 ], and entity normalization [ 6 ];
- search for language-model efficiency through few-shot learning [ 7 ], transfer-learning [ 8 ], prompting strategies [ 9 ], multimodal learning [ 10 ], or lightweight models [ 11 ];
- search for pieces of evidence in claim verification from scientific literature using specific language models [ 12 ] and RAG techniques [ 13 ];
- medical practice support: drug repurposing prediction [ 14 ], help for medical diagnoses [ 15 ], or detection of medical conditions [ 16 ].

These articles were then reviewed by external reviewers and the Yearbook editors, resulting in a selection of three articles presented at the annual meeting of the editorial board. The three articles selected are presented in greater detail in the appendix to this article:

- Andrade *et al.* [ 5 ] compared three approaches to manually annotate medical texts: the traditional selection of the whole span to be annotated from starting position to ending position, or the selection of a sub-part of the portion (lenient-span and point-wise methods);
- Liu *et al.* [ 13 ] produced a dataset (CoVERt) and a system (CliVER) for claim verification in clinical trials. They applied their model and compared three BERT models to identify a label among three types (support, neutral, refute);
- Molinet *et al.* [ 15 ] annotated a corpus of 314 clinical cases from the MedQA-USMLE corpus and they investigated how to generate fine-grained natural language explanations for correct and incorrect diagnoses.

## 3. Current Trends in Biomedical NLP

To study the current trends in 2024 publications, we first analyze the keywords provided by authors (Section 3.1). Second, we analyze the languages addressed (Section 3.2) and the sources of data exploited (Section 3.3). Then, we distinguish some trends in the use of language models (Section 3.4) and some issues on the tasks addressed (Section 3.5). Except for keywords, the analysis is made up of the top 350 citations according to the scores computed automatically.

### 3.1 Main Keywords Used in the Titles

As presented in Section 2.1.1, we collected much more papers this year than previous years. To illustrate the increase in the number of articles claiming to be concerned with NLP, we analyzed the distribution of some keywords in the titles of the articles collected since 2006. Figure 4 shows the number of articles per year whose title includes one of the main NLP-related keywords: generic terms (Natural Language Processing, NLP, Artificial Intelligence), and the terms related to data (Electronic Health Records, EHR, Social Media), approaches (BERT, Machine Learning), or application tasks (Named Entity Recognition, NER, Information Extraction).

**Figure 4.**
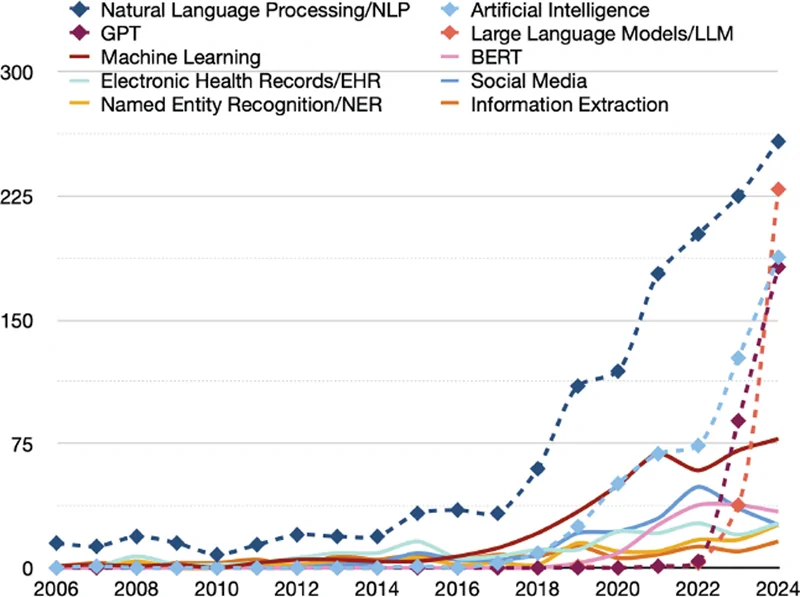
Number of articles per year whose title includes one of the main NLP-related keywords.

We can observe three main trends:

- a strong and continuous increase since 2017 in the use of “Natural Language Processing” or “NLP” (dashed dark blue line); this trend is similar for “Artificial Intelligence” (dashed light blue line), as if the two keywords were used as synonyms;
- an explosion in “Large Language Models” or “LLM” or “LLMs” (dashed pink line) and “GPT” (dashed purple line) since 2022, underlining the recent democratization of these new techniques;
- a moderate increase in other keywords, whether they refer to tasks (“Named Entity Recognition” or “NER” in yellow, “Information Extraction” in orange) or the data used (“Electronic Health Records” or “EHR” in light blue, “Social Media” in blue). These keywords are more emblematic of the usual work in NLP.

From the comparison between the first two and the last trends, it seems that the appropriation of language models by non-specialists in NLP leads to a huge increase in the number of related works and publications.

### 3.2 Languages Addressed

In the NLP works published in 2024, data in the English language are the most studied compared to data in other languages. In our opinion, there are two main reasons for that: (1) availability of huge volumes of datasets and reference annotations in English; and (2) availability of a great number of language models, both encoders and decoders, for the processing of English data. This makes work on English very attractive, as it permits to create new experiments and test various parameters quite easily. Then, Chinese continues to be the second most studied language. We have been observing this trend for several years, as testified by Figure 5 as well. After that, several active teams from specific countries work on datasets with their national languages, such as Dutch, French, German, Japanese and Korean. We also notice some works on languages that are less addressed, such as Danish, Italian, Persian, and Urdu. Overall, the languages addressed belong to various geographical areas (Europe, America, Asia), including some emerging languages like Urdu and Persian.

**Figure 5.**
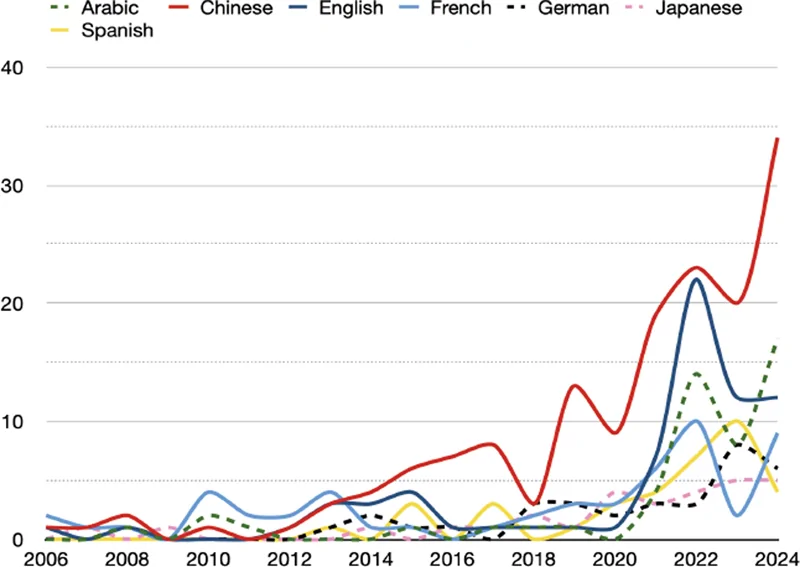
Number of articles per year whose title includes a language name (red line for Chinese, blue line for English, light blue line for French, yellow line for Spanish, dashed green line for Arabic, dashed pink line for Japanese, and dashed black line for German).

The following list is not exhaustive and only reflects a few topics addressed in each language among the top-scored publications:

- Arabic: multi-label classification model for medical questions [ 17 ];
- Chinese: NER [ 18 , 19 ], clinical decision-making in ophthalmology [ 20 ], encoding of traditional Chinese medicine knowledge with LLMs [ 21 ], dialog-based assistance for diagnosis [ 22 ], extraction of entities and relations [ 23 ], extraction of causality [ 24 ];
- Danish: identification of smoking status in patients [ 25 ];
- Dutch: identifying adverse drug reactions in electronic health records [ 26 ], International Classification of Diseases (ICD) coding [ 27 ], extraction of clinical concepts [ 28 ], sentiment analysis in social networks [ 29 ];
- French: detection of medical conditions of patients [ 16 ], de-identification of electronic health records (EHRs) [ 30 ], detection of adverse drug events in discussion forums [ 31 ];
- German: patient diagnosis [ 4 ], disambiguation of O2 supplementation in clinical narratives [ 32 ], annotation of medical concepts from SNOMED CT [ 33 ], detection of diagnosis in patients in a German-speaking Swiss hospital [ 34 35 ];
- Greek: conversational agent for hematologic malignancies [ 36 ];
- Italian: identification of delays in follow-up examination [ 37 ], NER [ 38 ];
- Japanese: fine-tuning and adaptation of various BERT models [ 39 ], detection of patient symptoms in clinical texts [ 40 ], quantification of semantic errors in incident reports [ 41 ];
- Korean: different text mining tasks with various language models [ 42 ], topic analysis of newspaper articles on nurse issues [ 43 ];
- Norwegian: deidentification of clinical texts [ 44 ];
- Persian: clinical spelling correction [ 45 ];
- Spanish: support for patients in understanding clinical trials and consent forms [ 46 ], clinical entity recognition [ 47 48 ], deidentification of EHRs [ 49 ];
- Swedish: topic modeling for COVID-19 vaccination [ 50 ];
- Urdu: NER [ 51 ].

Beyond the observations noticed for individual languages, we can indicate two other interesting trends. First, the availability of a variety of language models makes it easier to test and evaluate several such models for a given NLP task in a given language. This is the most frequent situation at the current timespan. Moreover, in addition to language models, researchers also evaluate traditional machine learning algorithms and rule-based approaches on a fairly systematic basis. Indeed, comparing the performance of different approaches for a given task, dataset and language occupies an important place. Second, the experiments are done on datasets in several languages, such as disambiguation of abbreviations in German, Portuguese and English [ 52 ], text mining in Korean and English [ 42 ], normalization of cross-lingual medical entities[ 6 ], semantic textual representations with LLMs and clinical knowledge graph with multilingual LLMs [ 53 ], or annotation of medical concepts from SNOMED CT in German and English [ 33 ]. In such works, the researchers may adopt various strategies: take advantage of multilingual models, exploit comparable monolingual models in the concerned languages, or combine one monolingual language model with machine translation.

Figure 5 summarizes the observations and shows the number of articles 3 per year whose title includes a language name. Because English dominates in terms of available resources and works carried out, it seems normal that this language name is seldom indicated in the titles (blue line), contrary to the other languages, which appear less endowed. As seen through the significant increase in the number of publications from China (Section 2.1.2), Chinese-related work or traditional Chinese medicine (red line) have been visibly increasing since 2012, and even more markedly since 2018. The increase in the number of articles mentioning another language (Arabic, French, German, Japanese, Spanish, etc.), especially in 2022, may be explained by the ability of language models to rapidly produce new resources and perform experiments in these languages.

### 3.3 Sources of the Data Processed

We observe interesting changes in the use of date sources processed in 2024. Overall, four main sources can be distinguished: clinical and health-related data, scientific literature, social media and newspapers.

The number of works, in which the processed data come from clinics and hospitals, increased very importantly in 2024. We assume that this trend is due to the availability of datasets like MIMIC, whose use is open to researchers [ 54 55 56 ], but it is mainly due to the availability of language models that can be deployed within hospitals and clinics [ 25 27 34 37 57 58 ]. This fact enables researchers to exploit privatized language models: researchers take advantage of these models while preserving the privacy of healthcare data. This trend also indicates that efficient collaborations between clinicians and computer scientists can be put in place, or that clinicians can master the exploitation of language models on their own. Non-clinical but health-related data are also exploited, such as reports on incidents in residential care facilities for the extraction of multiple incident factors due to medication [ 59 ] or the VAERS (Vaccine Adverse Event Reporting System) for signal management [ 60 ].

The scientific literature continues to be a popular datasource, due to its availability and the variety of information it contains. Hence, several works exploit it for various tasks, like recognition and normalization of biomedical named entities [ 61 ], biomedical RE [ 62 ], extraction of evidence and claims [ 13 ], and extraction of social and lifestyle factors [ 63 ].

Social media continue to attract researchers mainly because they shed some light on the interests and preoccupations of the population. Hence, social media are exploited for various questions and tasks, such as sentiment analysis [ 29 64 ], text classification with LLMs [ 65 ], extraction of adverse drug events [ 66 31 ], topic modeling [ 67 68 ], and information extraction on childhood health [ 69 ].

One new trend observed is related to the exploitation of newspaper articles. Hence, in one work newspapers are used for identifying family structures from obituaries [ 70 ], while in another work newspaper articles on nurse issues are exploited for topic analysis [ 43 ].

### 3.4 Language Models

As indicated in Section 3.1, in 2024 we observe an increased exploitation of language models and especially of the LLMs. Both, encoders [ 6 , 11 , 13 , 15 , 39 , 62 , 71 ] and decoders [ 3 , 7 , 15 , 72 – 74 ] are used by researchers for a wide variety of tasks. Among these tasks, we can mention: prediction of diseases [ 75 ] or cognitive decline [ 76 ] based on the content of clinical notes, emergency services (e.g., prediction of the risk of admission [ 77 ], surgery [ 78 ], or readmission [ 79 ]) and mining scientific literature (for instance to extract clinical knowledge on cardiac disorders [ 80 ]).

Besides, language models are often compared with other NLP approaches, such as traditional machine learning algorithms and rule-based systems. The comparison is done in terms of evaluation performance (precision, recall, accuracy, etc.), time needed, and carbon footprint.

### 3.5 NLP Tasks and Medical Domains

A great variety of well established tasks are addressed within the NLP area, which are related to information extraction (NER, relations, claims, etc.), summarization, simplification, coding, semantic similarity, etc. One new trend appeared due to the availability of multi-task language models, which motivates the tests on various tasks within the same experiment, provided that reference datasets are available in a given language [ 11 , 42 , 53 , 81 ]. Another trend, related to the huge need to have large amounts of annotated data, is the data augmentation task [ 18 , 19 , 65 ]. We assume that these trends will remain important for the NLP area.

We observe two main areas of medical activity where NLP is being applied: cancer and mental illness. For cancer, it mainly concerns breast cancer [ 82 83 ], lung cancer [ 84 85 ], prostate cancer, pancreatic cancer, neck cancer, and colorectal cancer. Beyond cancer, mental health remains at the heart of NLP-based research, either to predict a risk (depression [ 86 ], suicide) or the progression of a cognitive disease (Alzheimer's disease [ 87 ], dementia [ 88 ], etc.).

## 4. Conclusion

This synopsis paper presents the process we followed to identify the best papers for the NLP chapter of the IMIA Yearbook. We selected three scientific papers published in biomedical journals. These papers deal with various topics: manual annotation strategies in clinical data, claim verification in scientific literature, generation of explanations for diagnoses in clinical cases, and distillation of language models for various NLP tasks in English.

Among the main trends in the 2024 publications, we notice an interest to multi* frameworks, in which several languages, tasks or models are addressed within the same experiments. We assume this trend is due to the availability of language models and datasets. We also notice an intensive exploitation of clinical data, which is due to the capacity of clinicians to master these approaches and to collaborate with computer scientists. In addition to a more traditional use of clinical data, scientific literature and social media, we notice the emergence of newspapers as yet another source of information to be processed. In accordance with observations from previous years, there is an increasing number of publications issued from China and covering Chinese data, even if the processing of English data remains the most frequent. Finally, and not surprisingly, we observe a large adoption of LLMs for various tasks.

## Footnotes

1
https://pubmed.ncbi.nlm.nih.gov/

## Footnotes

2 We have included all European countries, with the exception of Russia, which is included in the “other countries” category.

## Footnotes

3 As returned by our Medline query (Section 2.1.1)
